# Re-exploring the requirement of dietary iodine intake in Chinese female adults based on ‘iodine overflow theory’

**DOI:** 10.1007/s00394-022-03065-w

**Published:** 2023-01-18

**Authors:** Yajie Li, Jun Wang, Xiaobing Liu, Weidong Li, Deqian Mao, Jiaxi Lu, Xiuwei Li, Hongxing Tan, Yanyan Liu, Junan Yan, Wei Yu, Chongzheng Guo, Xiaoli Liu, Xiaoguang Yang

**Affiliations:** 1 Changzhi Medical College, No. 360, Jiefang East Street, Changzhi, 046000 Shanxi People’s Republic of China; 2 School of Food and Drug, Shenzhen Polytechnic, Shenzhen, 518055 Guangdong People’s Republic of China; 3 Chinese Center for Disease Control and Prevention, National Institute of Nutrition and Health, Key Laboratory of Trace Element Nutrition of National Health Commission, No. 29, Nanwei Road, Beijing, 100050 People’s Republic of China; 4 National Reference Laboratory for iodine deficiency disorders, Chinese Center for Disease Control and Prevention, Beijing, 100050 People’s Republic of China; 5 Shenzhen Center for Chronic Disease Control, No. 2021, Buxin Road, Shenzhen, 518020 Guangdong People’s Republic of China

**Keywords:** Iodine, Incremental iodine intake, Incremental iodine excretion, Balance study, Chinese female adults, Iodine overflow

## Abstract

**Purpose:**

We re-explored the basal iodine requirement based on healthy Chinese female and a new iodine overflow theory was proposed for iodine balance study.

**Methods:**

Thirty-six Chinese healthy female adults (age 20.7 ± 1.1) were recruited for this study, which included 40 days low iodine depletion period and six stages of 30 days supplementation period. Uniform diets with low iodine were provided and the content of iodine in the diet was regulated by dairy products. The total iodine intake from food and the total iodine excretion through 24-h urine and staged feces were completely gathered and monitored. The incremental (Δ) intake and excretion over the range were calculated.

**Results:**

The iodine intake and excretion were 13.6 μg/day and 48.6 μg/day at the first stage, respectively. The incremental iodine intakes and excretions were 21.1 μg/day to 120.3 μg/day and 25.8 μg/day to 105.4 μg/day for the supplementation stages, respectively. According to the ‘iodine overflow theory’, the zero iodine balance (Δ iodine intake = Δ iodine excretion) derived from a mixed effect model indicated a mean iodine intake of 52.2 μg/d (1.0 μg/d kg). The RNI for iodine to healthy Chinese female adult was 73.1 μg/d (1.4 μg/d kg).

**Conclusion:**

A daily iodine intake of 52.2 μg/d may meet the basal iodine requirement for healthy Chinese female adults, and Chinese female may need more than 20% iodine intake than male based on the ‘iodine overflow theory’. The trial was registered at the Chinese Clinical Trial Registry in May 2018 (No: ChiCTR1800016184).

## Introduction

Iodine is an essential micronutrient for synthesis and release of the thyroid hormones [1]. Both iodine deficiency and excess will lead to thyroid disorders, i.e., it can be represented by a U-shaped curve between iodine intake and the occurrence of thyroid diseases [2].

Iodine deficiency will affect all stages of the life and cause a whole range of disorders termed iodine deficiency disorders (IDDs) when iodine intake is below a certain threshold [3]. During the past 20 years or so, universal salt iodization (USI) program is implemented to eliminate the hazard of iodine deficiency in China and this prophylactic strategy has achieved great success [4]. However, recent studies found that excess iodine intake have linked with adverse effects and disorders [5]. With the increasing thyroid diseases in the world, especially thyroid cancer, it is speculated that some relation may exist between the increasing of thyroid diseases and long-term USI program [6, 7]. Whether the iodine intake is too high and the recommended nutrient intake (RNI) of iodine is reasonable has aroused much concern. Therefore, establishing reasonable and reliable iodine dietary reference intakes (DRIs) for certain population is urgently necessary and can help people achieve a suitable iodine nutritional status.

The information provided by metabolic balance studies enables us to determine the minimal requirements of foodstuffs [8]. Because dietary iodine is rapidly and almost completely absorbed [9], the balance study could be directly used to determine iodine EAR of population by assessing the total iodine intake and excretion [10, 11]. However, because of ethical challenges, funding, complete dietary restrictions, and sample collection, only few studies on iodine balance have been carried out [12]. In China, there is a dearth of data on the iodine balance. At present, RNI of iodine for Chinese adult is 120 μg/day, which was determined according to the adjustment of adult body weight in US data [13]. However, some studies have found that Chinese adults have a lower tolerance to iodine than westerners [14, 15]. So, it is necessary to determine the RNI of iodine in Chinese adults.

In previous researches of traditional iodine balance, the dietary iodine intake dosage in these experimental diets was much higher than the current RNI level and these results showed that healthy subjects were in the negative iodine balance within the recommended intake range [10, 12, 16]. The calculated value will be higher when iodine intake is higher than the actual demand and the classical iodine balance experiment cannot also reasonably explain this phenomenon of ‘negative iodine balance”. Meanwhile, the normal iodine metabolism and iodine nutrition of Chinese residents have been greatly improved since the full implementation of USI in 1996. So, traditional analysis of balance study may not be suitable for Chinese individuals with iodine overnutrition, and a new iodine overflow theory was proposed [17, 18]. The ‘iodine overflow theory’ is: 1) healthy adults can absolutely and effectively utilize iodine, and when the iodine intake is sufficient for the body's metabolic needs and necessary storage, the excess iodine will be excreted; 2) iodine excretion increases with the increase of iodine intake and when iodine intake exceeds a certain threshold, the increased dietary iodine intake will be completely excreted, and this threshold should be the lower limit of iodine dietary reference intake for adults [18]. According to the overflow theory, we used the incremental iodine intake (Δ iodine intake) to assess the iodine requirement. In these previous studies, we found that the iodine requirement of Chinese adults may be lower than the current RNI [17, 18], and there may be differences in iodine requirement between male and female adults. Because these experiments lasted for a short time and the iodine intake range was small, which did not include the current RNI, the results need further confirmation.

To better evaluate the iodine requirement of Chinese female adults and verify the ‘iodine overflow theory’, we re-explored the iodine requirement of Chinese female adults based on the ‘iodine overflow theory’. In the present study, we extended the experimental time, which included the female menstrual cycle, and increased the range of iodine intake. Our research will provide the basic data for formulating the suitable RNI of iodine for Chinese adults and also offer a new calculation way of iodine requirement.

## Materials and methods

### Sample size

The sample sizes were calculated by G*Power (version 3.1.9). As the study was a before–after self-controlled research, it was estimated that 34 participants would be required for a paired *t *test to detect a medium effect size (*F* = 0.5) with a statistical power (1–β error probability) of 80% and an *α* of 0.05. After comprehensively considering factors such as funding, sample collection, comparability, etc., 36 subjects were determined.

### Subjects

A total of 36 healthy female volunteers aged 19–23 years were recruited from the students of Changzhi Medical College to participate in this study. Before the experiment, 24-h urine samples were collected to determine the baseline iodine nutritional status of subjects. All of the volunteers were subjected to a physical examination and provided morning urine sample and fasting blood samples used for screening. A questionnaire was used to evaluate the disease history and dietary habits of the subjects. Height was measured to the nearest 0.1 cm using a stadiometer, and weight was measured to the nearest 0.1 kg using an electronic scale. Thyroid hormones, the indicators of liver and kidney function, such as thyroid stimulating hormone (TSH), free thyroxine (FT4), free triiodothyronine (FT3), glutamic pyruvic transaminase (ALT), and glutamic oxaloacetic transaminase (AST), were determined. TSH, FT4, and FT3 were measured using electrochemiluminescence immunoassays (COBAS 601 analyzer, Roche Diagnostic, Switzerland). ALT and AST were determined by automatic blood biochemical analyzer (COBAS INTEGRA400 Plus). Those with thyroid disorders, abnormal liver and kidney function or used drugs for thyroid diseases were excluded from this study. All of the volunteers were considered to be healthy and had no medication history of iodine in the past 2 weeks.

The trial was registered at the Chinese Clinical Trial Registry (No:ChiCTR1800016184) and approved by the Ethical Committee of the National Institute for Nutrition and Health, Chinese Center for Disease Control and Prevention(Ethical Approval Number:2018–001). The purpose and procedure of this study was carefully explained to all the subjects, and informed consent was obtained from each participant.

### Experimental design

USI has been carried out in Shanxi province for more than 20 years. Through the baseline on urine iodine concentration in female students, we found that the urine iodine of most female students exceeded the optimal iodine nutrition according to the related standard [19]. To eliminate the state of iodine supersaturation, there was a depletion period of low iodine before the formal experiment. This study started in March and ended in June, including low iodine depletion period and dietary iodine supplementation period. According to the dynamic monitoring of female students' urinary iodine excretion, the low iodine depletion period lasted for 40 days. During this period, the meal without iodized salt and purified water without iodine was provided to the subjects. 24-h urine samples were collected and 24-h urine iodine concentration (UIC) was measured by Quantitative Test Kit AR for Urinary Iodine (Wuhan Zhongsheng, China) to monitor the iodine excretion of subjects from the seventh day of low iodine period. After a 40-day low iodine period, according to the increasing of dietary iodine intake, the iodine supplementation period was divided into six stages, each stage lasting 5 days, and the intake of dietary iodine increased by 0, 20, 40, 60, 90, and 120 μg at each stage, respectively.

**Fig. 1:**
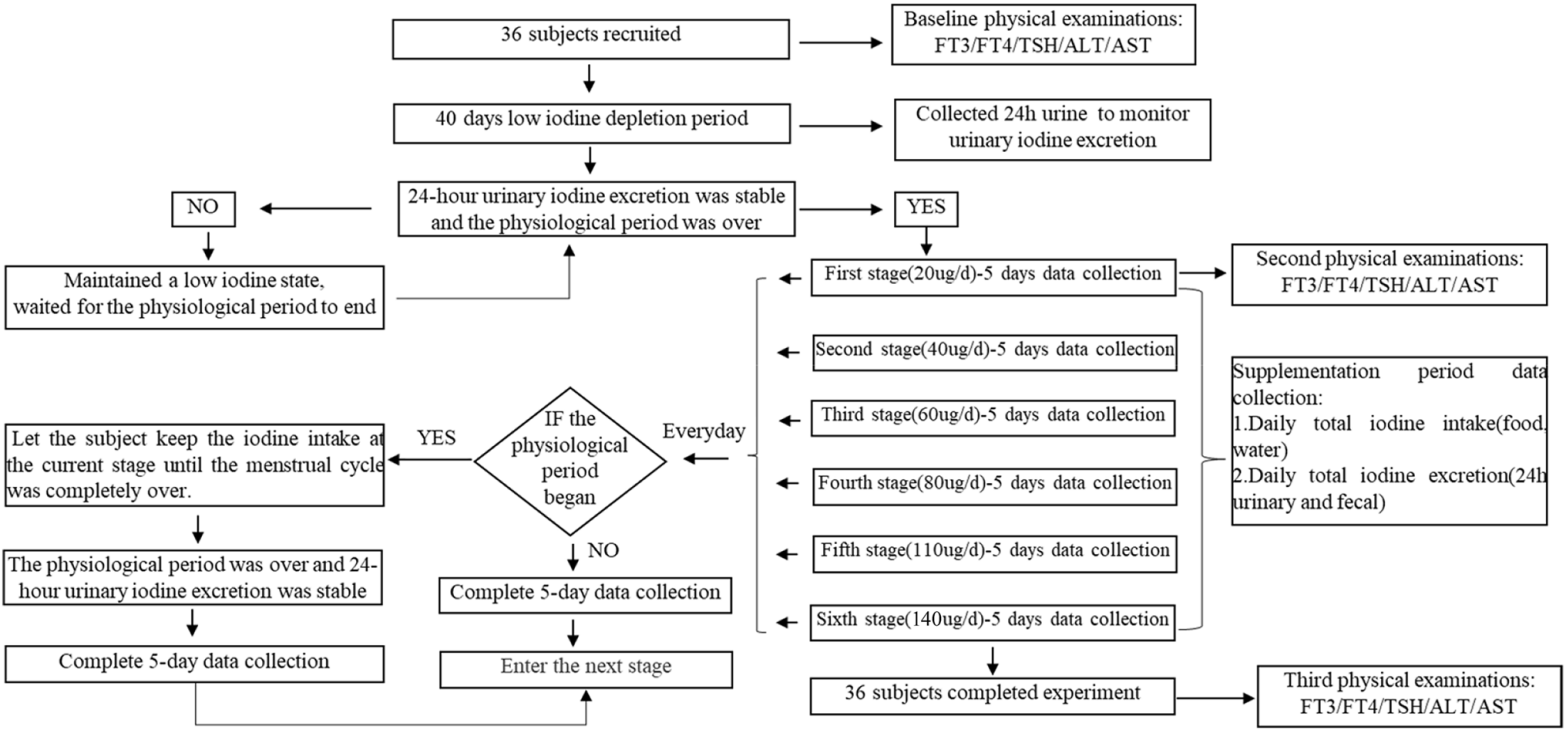
Experimental flow chart

To reduce the error of menstrual iodine loss, female volunteers began to enter the dietary iodine supplement period after the low iodine depletion period ended and the menstrual cycle ended in April. During dietary iodine supplementation period, when the menstrual cycle of the subject began, we recorded and let the subject keep the iodine intake at the current stage until the menstrual cycle was completely over. We also used the Quantitative Test Kit AR for Urinary Iodine to ensure the urine iodine of the subject was stable after the menstrual cycle, and then continued the subsequent experimental stage. The study flow chart is shown in Fig. 1.

### Experimental diet and sample collection

Considering the influence of iodine in food, before the experiment, the raw materials and condiments were all sampled and measured in advance and high iodine of food were excluded. In this study, non-iodized salt was provided in all the meals and purified water without iodine was used for drinking and cooking. Non-iodized salt and purified water were also sampled and measured to ensure that there was no iodine. According to the basic data of the typical dietary pattern obtained from the China National Nutrition and Health Survey 2010–2013 (CHNNS 2010–2013) and market supply, the foods in the experimental diet were assorted to design a 5-day cycle recipe. The food met the principles of nutrient balance, and the macronutrients and micronutrients in the diet were as consistent as possible at all stages. The macronutrient in diet could meet the needs of energy demand, which is shown in Table 1. The dietary iodine content provided by the recipe was basically the same every day. All the food was uniformly prepared in a standardized canteen. The iodine content in different stages was controlled by the incremental amount of dairy products. The iodine content of dairy products was measured in advance, and dairy products were given at each stage by accurate weighing to increase iodine intake. Subjects were asked to consume the food and fruits, and to leave residual food in the table ware which could accurately estimate actual daily consumption every day. All the volunteers were requested to drink purified water without iodine. So, the total iodine intake was the dietary iodine intake. During the trial, subjects not allowed to consume food or drink other than those specified and water. A duplicate dietary sample was collected, and then each food sample were weighted, homogenized, and preserved in the freezer at –20℃ until further analyses.

**Table 1:** Intake of main dietary nutrients in basic diet

Characteristic	Actual intake (g/d)	Energy (%)
Carbohydrate	313.0 ± 55.1	67.1 ± 2.7
Protein	66.9 ± 12.2	14.4 ± 1.6
Fat	37.9 ± 7.9	18.3 ± 1.9
Iodine*	13.6 ± 6.2	–

Nor measured

^*^ (μg/d)

### Urine and fecal specimen collection and measurement

All the subjects were asked to collect the completed 24-h urine with a clean cup and portable plastic urine bag and the amount of urine missed or spilled by each subject every day was recorded. The sampling started from 8:00 am of the day to 8:00 am the next day. 24-h specimens of urine was weighed and recorded once the 24-h collection was completed. Subsamples of 24-h urine specimens (approximately 10 ml of each) were sealed and stored at –20℃ in the freezer. Urine specimens were evaluated for completeness based on the criteria: (1) creatinine excretion < 0.1 mmoL Kg/body weight day, (2) 24-h urine volume < 500 ml. If any one of the above criteria applied, the 24-h urine collection was deemed incomplete.

Similar to urine sample collection, fecal specimens were completely collected with a portable plastic bag every day. Before the start of each stage and at the end of sixth stage, capsules of carmine red dye were given to subjects before breakfast to determine the initiation and end of feces collection. All the fecal samples for each subject at the same stage were weighted and homogenized. An aliquot of fecal specimens (approximately 200 g of each) were lyophilized and preserved in the freezer at −20℃. Finally, the subsamples of 24-h urine specimens and the aliquot of fecal specimens were transported to the National Institute for Nutrition and Health, Chinese Center for Disease Control and Prevention. The iodine content in urine and feces was measured by inductively coupled plasma mass spectrometer (ICP-MS) with a modified Sandell–Kolthoff method after acid wet washing of samples [20]. The total daily iodine excretion was assessed by complete collection urine and feces specimens during each stage.

### Quality control

To ensure the quality of the test, we have taken a series of control measures. Before the experiment, to avoid using high-iodine raw materials and condiments, all of them were all sampled and measured in advance. The staple food and non-staple food of each meal were kept separately, and each food could not be mixed when eating. The intake of each food was accurately weighed and recorded. Portable urine bags for the subjects were provided to collect urine and the volunteers were reminded to complete the collection of all urine in every day. The amount of urine missed or spilled by each subject every day was recorded and, during the experiment, subjects were asked to move only around the campus. Data that did not meet the standards through urine sample integrity analysis were excluded. Quantitative Test Kit AR for Urinary Iodine (Wuhan Zhongsheng, China) was used to monitor 24-h urine iodine concentrations, which could reflect the compliance of subjects. If the results of urine iodine were abnormal, the subjects got immediate feedback and were reminded to control diet. Capsules of carmine red dye were used to ensure the integrity of fecal samples. Three physical examinations at professional hospital during the whole process of the project were conducted to ensure the volunteers' health and normal thyroid function, including the baseline, the end of the first stage, and the end of the experiment.

### Statistical analysis

We used EXCEL (2016; Microsoft), SPSS statistics software (version 19.0), and R statistical programming environment (version 3.5.1) by use of the packages nlme and ggplot 2 for data processing and analysis. Data were examined for normality by use of the Shapiro–Wilk test. Descriptive data were expressed as means ± SD or medians with interquartile ranges. Statistical significance was defined at *p* < *0.05*.

Each subject provided 5-day data at each stage, including iodine intake data from diet and iodine excretion data from urine and feces. The data on incremental iodine intakes (Δ iodine intake by diet) were obtained by the daily dietary iodine intake from the second stage to the sixth stage minus the corresponding iodine intake at the first stage. The calculation of incremental iodine excretion (Δ iodine excretion by urine and feces) was obtained by the iodine excretion for urine and feces from the second stage to the sixth stage minus the corresponding iodine excretion at the first stage. Data on the incremental iodine retention (Δ iodine retention) were calculated for each subject as difference between measured Δ iodine intake and measured Δ iodine excretion. Data on Δ iodine intake, Δ iodine excretion, and Δ iodine retention were expressed in μg/day, as well as in μg/kg bw day.

The dose–response relation between Δ iodine intake and Δ iodine excretion was assessed by fitting the mixed effects models (MEMs) for the micrograms per day and micrograms per kilogram of body weight per day. The individuals’ Δ iodine intake was defined as a fixed factor and the individuals were defined as a random factor. The final model selection was based on Akaike information criterion (AIC) and was also evaluated by using goodness-of-fit plots. The agreement between the observed and the predicted values for Δ iodine excretion and Δ iodine retention was evaluated by use of Pearson’s correlation coefficient. Zero balance (Δ iodine retention = Δ iodine intake − Δ iodine excretion, Δ iodine retention = 0 μg/day) was obtained from the MEMs of Δ iodine intake compared with Δ iodine retention micrograms per day and micrograms per kilograms per day, which was used to present the estimated average requirement (EAR). In the balance studies, due to the characteristics and complexity of its design and calculations, the recommended nutrient intake (RNI) for iodine is set using a CV of 20% of the EAR (therefore, for iodine the RNI is 140% of the EAR) [13].

## Results

### Baseline data of the subjects

A total of 36 Chinese young female adults aged 20.7 ± 1.1 were enrolled in this experiment. Physical examinations were carried out to ensure the healthy status of subjects during the study. Anthropometric measurements of the subjects during the three period are described in Table 2.

**Table 2:** Subject’s basic characteristics

Characteristics	Values at baseline	Values at the end of first stage	Values at the end of experiment
*N*	36	36	36
Gender	Female	Female	Female
Age(Years)		20.7 ± 1.1	
Height(cm)		159.6 ± 5.8	
Weight(kg)	53.7 ± 8.2	53.0 ± 7.6	52.6 ± 7.5^a^
BMI(kg/m^2^)	21.1 ± 3.0	20.7 ± 2.9^a^	20.6 ± 2.8^a^
UIC(μg/L)	233.3 ± 122.5	20.37 ± 9.1^a^	89.4 ± 28.3^ab^
Urine volume(L)	1.2 ± 0.5	1.9 ± 0.7^a^	1.6 ± 0.5^a^
24-h UIE(μg)	242.1 ± 106.7	35.3 ± 12.3^a^	138.3 ± 37.7^ab^
ALT(U/L)	12.6 ± 5.5	13.0 ± 5.1	11.8 ± 4.6
AST(U/L)	16.4 ± 3.6	17.4 ± 3.0	17.3 ± 3.5
Creatinine(μmol/L)	56.6 ± 6.0	56.9 ± 5.8	60.7 ± 5.8^ab^
Uric acid(μmol/L)	277.8 ± 50.9	266.9 ± 47.8	260.8 ± 46.8^a^
TSH(mIU/L)	2.4 ± 1.0	1.2 ± 0.4^a^	2.2 ± 0.8^b^
FT4(pmol/L)	11.6 ± 1.50	10.6 ± 1.4^a^	10.0 ± 1.5^ab^
FT3(pmol/L)	5.6 ± 0.4	4.8 ± 0.6^a^	4.9 ± 0.5^a^
Serum iodine(μg/L)	68.0 ± 10.0	57.1 ± 11.0^a^	58.9 ± 13.3^a^

Values are expressed as mean ± SD (ranges); differences were examined by repeated measure ANOVA by use of Bonferroni correction for multiple comparisons in the study

^a^The mean value is significantly different from that obtained at baseline (*P* < 0.05)

^b^The mean value is significantly different from that obtained at the end of first stage (*P* < 0.05)

All subjects were in good health, and thyroid hormones were at normal level. Some biochemical markers had changed in the whole period of experiment. Significant differences were observed in weight, body mass index (BMI), UIC, urine volume, urinary iodine excretion (UIE), creatinine, uric acid, TSH, FT4, FT3, and serum iodine (*p* < *0.05*). However, all values were within the normal range and there was no response for adverse health effects in this study.

### Urinary iodine concentration and excretion level in this study

**Fig. 2:**
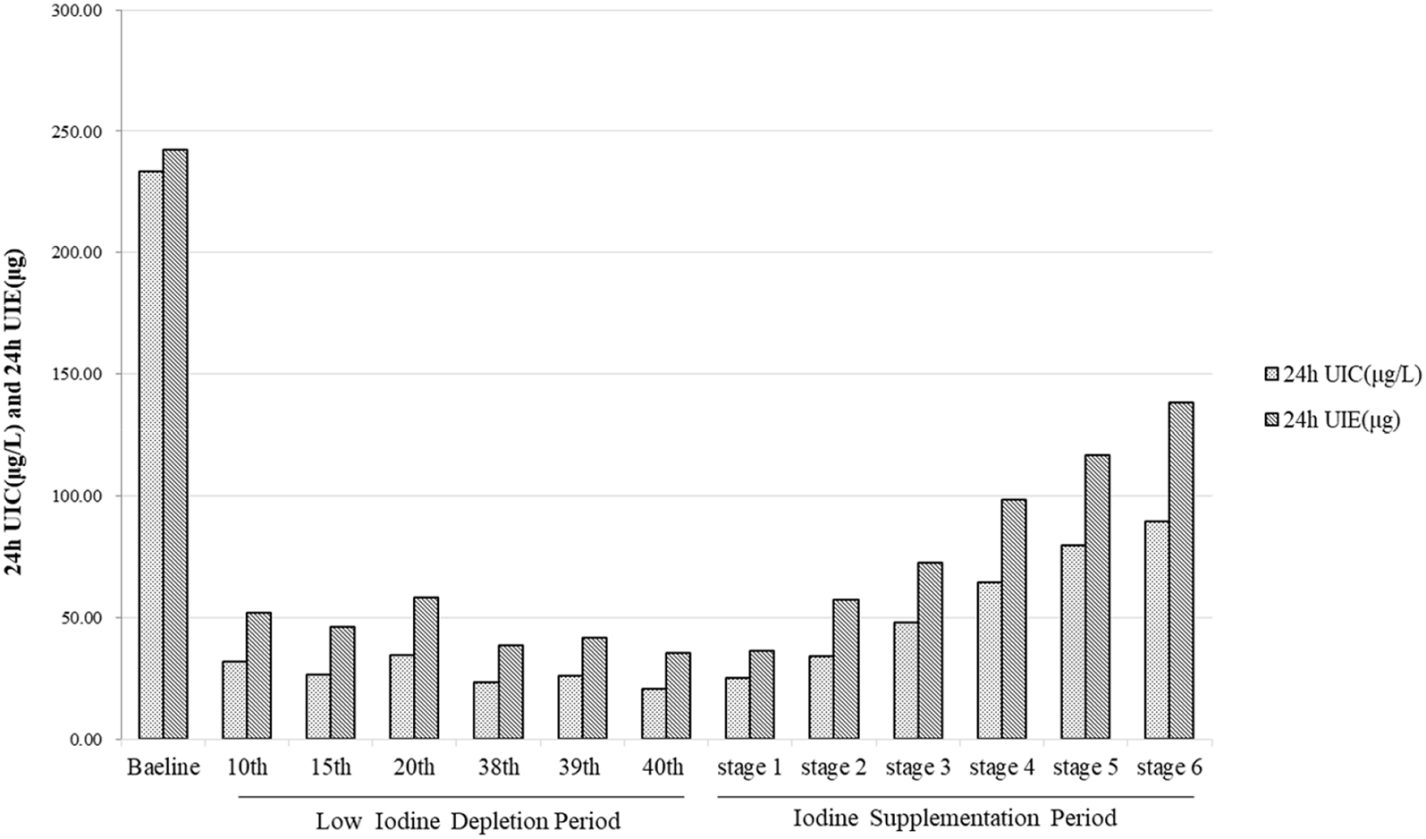
24-h urinary iodine concentration level (μg/L) and 24-h urinary iodine excretion level (μg)

UIC can reflect very recent iodine nutrition. At baseline, the 24-h UIC was 233.3 ± 122.5 μg/L and 24-h UIE was 242.1 ± 106.7 μg/day, which indicated an iodine overnutrition for the majority of subjects. From the seventh day of low iodine period, we collected 24-h urine from all subjects and monitored UIC. As shown in Fig. 2, the 24-h UIC and 24-h UIE of subjects in the depletion period gradually decreased. After 40 days of depletion period, 24-h UIC and 24-h UIE basically plateaued and the average 24-h UIC and 24-h UIE dropped to 20.4 μg/L and 35.3 μg/day. At the end of first stage, all subjects reached the final stage of low iodine; at this time, the thyroid hormone (TSH, FT3, FT4) and serum iodine concentration of all subjects decreased, but all indexes were within the normal range, indicating that the thyroid function of all subjects was still in normal state. After the low iodine period, the 24-h UIC and 24-h UIE were gradually increased with the dietary iodine intake at each stage.

### Association between iodine intake and excretion or retention across dosages

In our study, 1,080 data points were initially collected, which came from dietary iodine intake, iodine excretion by urine and feces. Based on the urine exclusion criteria and outlier for Δ iodine intake, Δ iodine excretion, and Δ iodine retention, 225 data points were removed, leaving 855 data points for data analysis. The observed and predicted iodine intake, iodine excretion, and iodine retention expressed as μg/d and μg/d kg are shown in Table 3.

**Table 3:** Observed and predicted iodine intake, iodine excretion, iodine retention, and incremental iodine intake, excretion, and retention for 36 young female adults^1^

	Stage 1	Stage 2	Stage 3	Stage 4	Stage 5	Stage 6
Observed, μg/d						
Iodine intake	13.6 ± 6.2	34.1 ± 7.6	53.7 ± 7.5	74.4 ± 7.3	101.7 ± 7.1	133.0 ± 6.5
Iodine excretion	48.6 ± 14.6	72.9 ± 18.7	85.8 ± 19.5	103.2 ± 24.3	127.7 ± 25.3	154.1 ± 29.7
Iodine retention	−34.9 ± 15.3	−38.8 ± 19.0	−32.1 ± 20.4	−28.8 ± 25.2	−26.0 ± 24.0	−21.2 ± 29.6
ΔObserved, μg/d						
ΔIodine intake	–	21.1 ± 8.4	39.6 ± 8.4	60.9 ± 9.3	88.2 ± 8.7	120.3 ± 7.8
ΔIodine excretion	–	25.8 ± 16.3	37.0 ± 18.4	54.7 ± 25.7	77.6 ± 28.2	105.4 ± 31.2
ΔIodine retention	–	–4.7 ± 17.2	–2.6 ± 19.9	6.2 ± 27.0	10.6 ± 27.8	14.8 ± 31.9
Predicted^2^, μg/d						
Iodine intake	–	n.a	n.a	n.a	n.a	n.a
Iodine excretion	–	25.0 ± 7.0	39.4 ± 7.3	56.0 ± 8.9	77.2 ± 9.9	103.0 ± 11.2
Iodine retention	–	–4.0 ± 2.8	0.3 ± 4.1	4.8 ± 5.7	11.0 ± 7.5	17.2 ± 10.2
Observed, μg/d kg						
Iodine intake	0.3 ± 0.1	0.7 ± 0.2	1.0 ± 0.2	1.5 ± 0.2	2.0 ± 0.3	2.6 ± 0.3
Iodine excretion	0.9 ± 0.3	1.4 ± 0.4	1.6 ± 0.4	2.0 ± 0.5	2.5 ± 0.5	3.0 ± 0.6
Iodine retention	–0.7 ± 0.3	–0.7 ± 0.4	–0.6 ± 0.4	–0.6 ± 0.5	–0.5 ± 0.5	–0.4 ± 0.6
Predicted^3^, μg/d kg						
Iodine intake	–	n.a	n.a	n.a	n.a	n.a
Iodine excretion	–	0.5 ± 0.1	0.8 ± 0.2	1.1 ± 0.2	1.5 ± 0.2	2.0 ± 0.3
Iodine retention	–	–0.1 ± 0.1	0.0 ± 0.1	0.1 ± 0.1	0.2 ± 0.2	0.3 ± 0.2

^1^Values are expressed as means ± SDs. *N* *a*. not applicable

^2^Predicted excretions were calculated from the observed intakes by use of MEM: Δ iodine excretion (μg/day) = 0.782 × Δ iodine intake (μg/day) + 8.414. Predicted retentions were calculated from the observed intakes by use of MEM: Δ iodine retention = 0.218 × Δ iodine intake (μg/day)–8.414

^3^ Predicted excretions were calculated from the observed intakes by use of MEM: Δ iodine excretion (μg/d kg) = 0.782 × Δ iodine intake (μg/d kg) + 0.161. Predicted retentions were calculated from the observed intakes by use of MEM: Δ iodine retention (μg/d kg) = 0.218 × Δ iodine intake (μg/d kg) –0.161

**Fig. 3:**
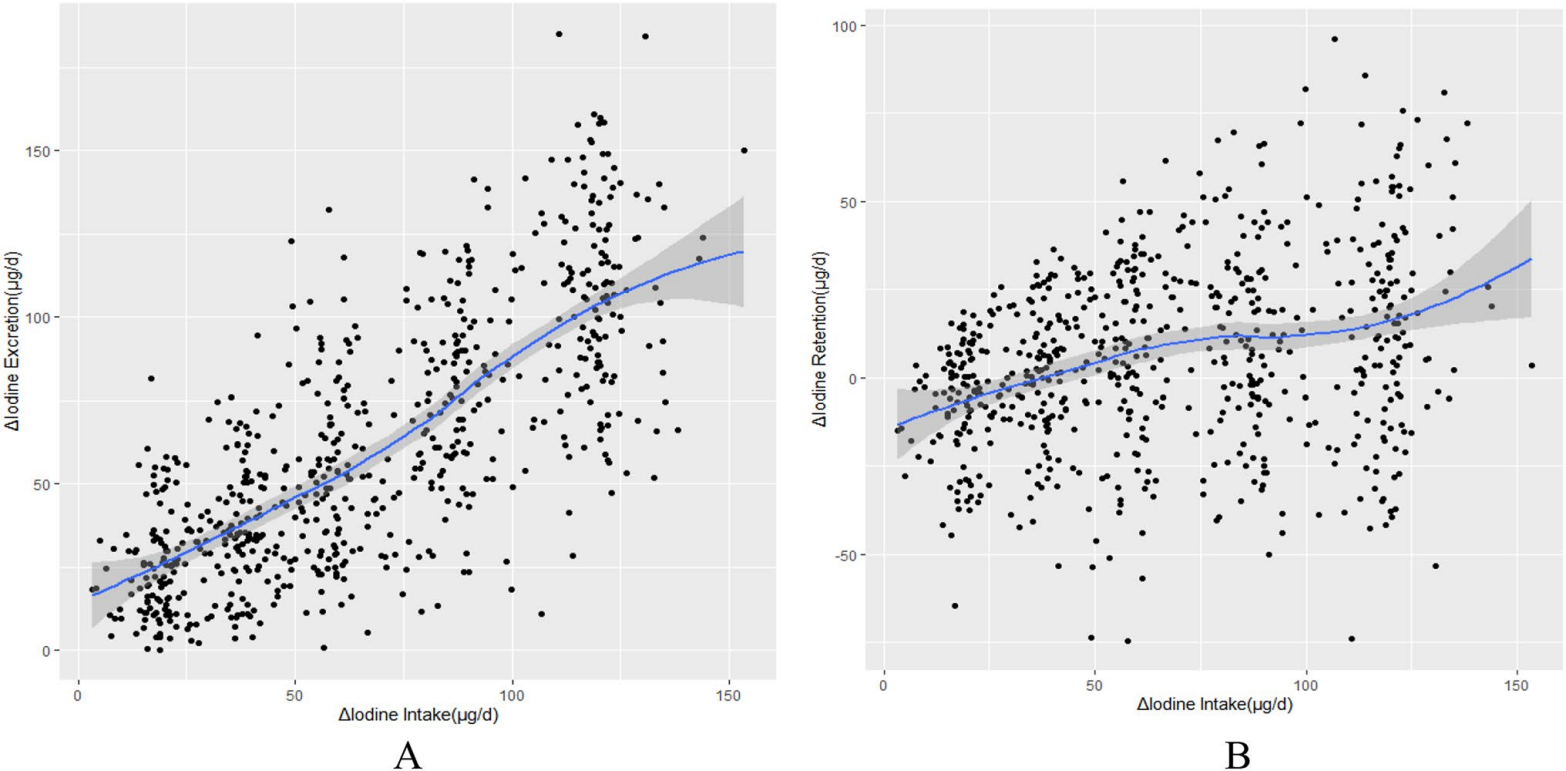
Association between Δ iodine intake and Δ iodine excretion (μg/d) (**A**) and Δ retention (μg/d) (**B**). The filled line and a light gray area represent the fitted line and the corresponding 95% CI. A: Δ iodine excretion (μg/d) = 0.782 × Δ iodine intake (μg/d) + 8.414; B:Δ iodine retention(μg/d) = 0.218 × Δ iodine intake (μg/d)–8.414

**Fig. 4:**
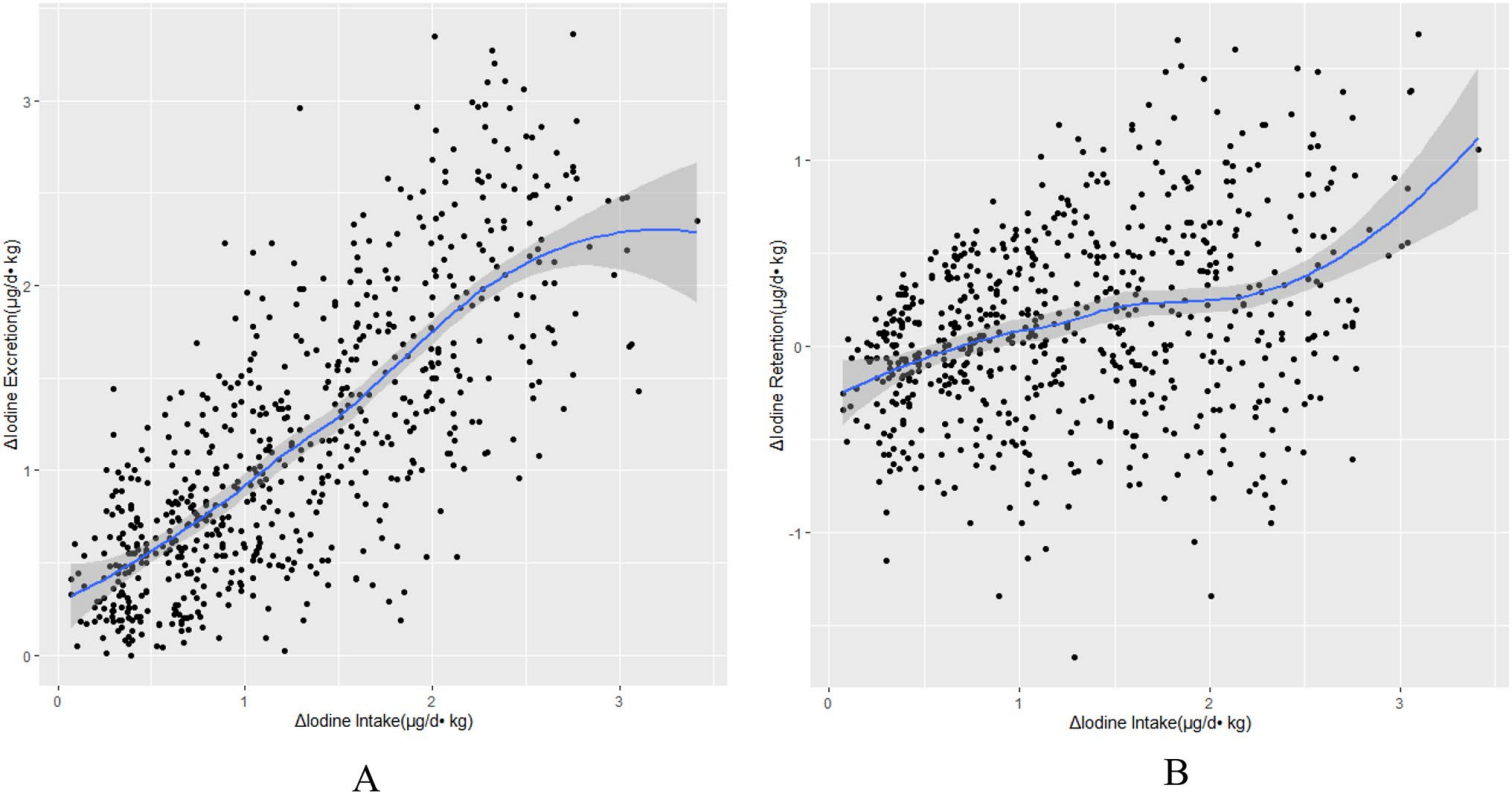
Association between Δ iodine intake and Δ iodine excretion (μg/d kg) (**A**) and Δ retention (μg/d kg) (**B**). The filled line and a light gray area represent the fitted line and the corresponding 95% CI. A: Δ iodine excretion (μg/d kg) = 0.782 × Δ iodine intake (μg/d kg) + 0.161; B: Δ iodine retention (μg/d kg) = 0.218 × Δ iodine intake (μg/d kg) –0.161

In this study, iodine intake in each stage was 13.6, 34.1, 53.7, 74.4, 101.7, and 133.0 μg/d, respectively. It increased in turn. Meanwhile, iodine excretion (48.6–154.1 μg/d) also increased in each stage. From stage 1 to stage 6, the iodine intake was lower than the iodine excretion, the iodine retention was negative, and all subjects were in negative iodine balance. The changing trends for iodine intake, iodine excretion, and iodine retention expressed as μg/d kg were the same. The MEMs were used to predicted dose–response association between Δ iodine intake and Δ iodine excretion and between Δ iodine intake and Δ iodine retention, respectively. The predicted data for Δ iodine excretion and Δ iodine retention increased from stage 2 to stage 6. The predicted Δ iodine retention gradually became positive from the third stage, which meant the inflection point could be between stage 2 and stage 3. There was a liner dose–response relation between the Δ iodine intake and Δ iodine excretion (Figs. 3A, 4A). We also observed an association between Δ iodine intake and Δ iodine retention (Figs. 3B, 4B).

The predicted dose–response association between Δ iodine intake and Δ iodine excretion obtained from MEMs agreed well with the observed data. There was a strong correlation between the observed and predicted data for Δ iodine excretion (*r* = 0.788, *p* < 0.001 for μg/d data) and Δ iodine retention (*r* = 0.461, *p* < 0.001 for μg/d data). It represented a good result of the model to predict the iodine excretion and retention from iodine intake.

The association between Δ iodine intake and Δ iodine excretion was described as following equations:

1 $$\Delta {\text{ iodine excretion }}\left( {\mu {\text{g}}/{\text{d}}} \right) \, = \, 0.{782} \times \, \Delta {\text{ iodine intake }}\left( {\mu {\text{g}}/{\text{d}}} \right) \, + {8}.{414}$$

2 $$\Delta {\text{ iodine excretion }}\left( {\mu {\text{g}}/{\text{d kg}}} \right) \, = 0.{782} \times \, \Delta {\text{ iodine intake }}\left( {\mu {\text{g}}/{\text{d kg}}} \right) \, + \, 0.{161}$$

The association between Δ iodine intake and Δ iodine retention was described as following equations:

3 $$\Delta {\text{ iodine retention }}\left( {\mu {\text{g}}/{\text{d}}} \right) \, = \, 0.{218} \times \, \Delta {\text{ iodine intake }}\left( {\mu {\text{g}}/{\text{d}}} \right) \, {-}{ 8}.{414;}$$

4 $$\Delta {\text{ iodine retention }}\left( {\mu {\text{g}}/{\text{d kg}}} \right) \, = 0.{218 } \times \, \Delta {\text{ iodine intake }}\left( {\mu {\text{g}}/{\text{d kg}}} \right) \, {-}0.{161}$$

When Δ iodine balance was reached, Δ iodine intake = Δ iodine excretion and Δ iodine retention = 0 μg/d; at this time, it could be calculated by the first or third equations that the Δ iodine intake was 38.6 μg/d. The data on incremental iodine intakes (Δ iodine intake) were obtained by the daily dietary iodine intake from the second stage to the sixth stage minus the corresponding iodine intake at the first stage. According to the iodine intake of stage 1 (13.6 μg/d), the lower limit of iodine demand was 52.2 μg/d (1.0 μg/d kg), i.e., the level of the EAR was 52.2 μg/d (1.0 μg/d kg).

In the balance studies, the RNI of iodine is 140% of the EAR [13]. According to this criterion, the calculated iodine RNI was 73.1 μg/d (1.4 μg/d kg).

## Discussion

In our study, we explored the iodine requirement in Chinese female adults using iodine balance technique. According to the ‘iodine overflow theory’, results showed that the minimum daily iodine intake was 52.2 μg/d (1.0 μg/d kg) in Chinese female adults. Iodine intake once exceeded 52.2 μg/d, iodine intake could maintain normal thyroid hormone synthesis, which was the lower limit of iodine demand for Chinese female adults. The results for RNI was suggested as 73.1 μg/d (1.4 μg/d kg).

EAR and RNI are important references of certain nutrients, and they can help people achieve an appropriate nutritional status. Since it is based on particular age, sex, and physiological condition, it varies in different countries and regions. Based on the turnover studies and ^131^I radioiodine accumulation data [16, 21, 22], a daily iodine intake of 95 μg and 150 μg is defined as the adult iodine EAR and RNI in the United States [23] and Canada. Australia and New Zealand determined that the value of EAR and RNI is 100 μg/d and 150 μg/d, respectively [24]. With a total intake of 100 μg/d, the iodine in the thyroid gland could reach appropriate nutritional status, and the European Scientific Committee for Food recommended the EAR and RNI of iodine as 100 and 130 μg/d, respectively. However, due to lack of data on the iodine balance, China used research results from other countries to indirectly determine RNI. The EAR of iodine was 85 μg/d, and the RNI was suggested as 120 μg/d.

The present study calculated the EAR and RNI as 52.2 μg/d and 73.1 μg/d for Chinese female adults. It was lower than the data found in western countries. Compared with previous studies in China, this result was also lower than the data of China [10]. This difference might be caused by some reasons.

First, since the full implementation of USI in 1996, China has completely eliminated iodine deficiency, and the iodine nutrition of residents has been greatly improved. Therefore, the normal iodine metabolism and iodine nutrition of Chinese residents will inevitably change accordingly. Meanwhile, some studies have found that the tolerable upper intake level (UL) in Chinese adults was lower than western adults [14, 15], which meant different populations with different iodine nutritional status had different metabolism and requirement of iodine. Under normal conditions, the clearance rate of kidney is relatively constant in adults [25], so UIC is a sensitive index reflecting iodine intake in recent days. Median urine iodine concentration (mUIC) is recommended for assessing iodine nutrition at population level [26]. Daily iodine intake can be extrapolated from urinary concentration [23]. A urinary iodine concentration of 100 μg/L corresponds to about 150 μg of iodine per day in adults [27]. If the current urine iodine standard overestimates after long-term USI in China, it follows that the related recommended daily iodine intake may also be overestimated for the current Chinese population. According to an iodine nutrition survey from 1994–1995 in Shanghai, the mUIC of 2,640 students aged 9–13 years was 64.50 μg/L [28]. This population might be slightly iodine deficient with the current standard, but the rate of goitre by palpation was only 1.55% and the children’s intelligence quotient (IQ) did not harm. Our previous study [29] found after 20 years of implementing USI, Chinese pregnant women with a mUIC of 107.4 μg/L, lower than the WHO’s 150 μg/L, could maintain normal thyroid function in both themselves and their newborns. It also indicated that the iodine requirements of the Chinese population may be lower than the existing recommended standards.

Second, study on iodine balance is one of the classic, efficient, and most commonly used methods for determining iodine RNI [10, 11]. Many countries have established iodine RNI of adults according to experimental data of iodine balance studies. But the dietary iodine intake dosage in these experimental diets was much higher than the current RNI level. Dworkin et al. [12] had reported a study on iodine balance in pregnant women, most of them were in negative iodine balance and the iodine intake was 80–250 μg/d. Vought [16] had also reported that, in their iodine balance studies, a volunteer showed positive iodine balance when iodine intake was 606.5 μg/d. In the experiment of iodine balance in healthy Chinese women performed by Tanlong et al.[10], the iodine intake was 173.5 μg/d and 467.3 μg/d, which were much higher than the 120 μg/d. The calculated value will be higher when iodine intake is higher than the actual demand.

Finally, the iodine metabolism state of healthy adults should be “zero balance” or “positive balance”, and the corresponding iodine intake at “zero balance” is the EAR of the population [30]. All subjects in this study were in negative iodine balance throughout the different iodine stages according to traditional iodine balance. This result is consistent with previous studies in adults [21, 31]. Dworkin et al. [12] had reported that 77 out of 129 cases were in negative iodine balance in their iodine balance study. Malvaux et al. [31] and Vought et al. [16] had also reported that a majority of volunteers were in negative iodine balance in their studies. It is impossible for healthy adults to maintain ‘negative iodine balance’ constantly, and the classical iodine balance experiment cannot reasonably explain this phenomenon. Interestingly, when we used the ‘iodine overflow theory’ and re-analyzed the data, we found that the inflection point could be between the stage 2 and stage 3, and the predicted Δ iodine retention gradually became positive from the third stage. After the long-term USI, people are in a state of iodine overnutrition, the classic iodine balance study may not be suitable for individuals with iodine overnutrition. The EAR cannot be calculated from negative iodine balance, the method of iodine overflow could be used to determine the lower limit of iodine requirement for young adults. It does not require the population to reach zero iodine balance, which to some extent makes up for the defect that the iodine balance method cannot evaluate the iodine requirement in the case of negative iodine balance. Our method may open up a new way to develop the dietary reference intake of iodine for healthy adults even if they are in negative iodine balance.

In this study, we spent more than 2 months and set up six dosage stages and each stage had 5 days, the iodine intake ranged from 13.6 to 133.0 μg/d, which included the daily recommended intake of healthy adults. Strict quality control was used to ensure iodine intake and the integrity of sample collection. The experiment included female menstrual periods, with consistent iodine diet and urine iodine monitoring, and the influence of female menstrual periods on the results was avoided. After excluding some data, the final number of data points was 855, which was larger than the sample size of the Dwokin’s et al. [12] recommendation for a minimum of 129 metabolic test data points. The results of this study could ensure accuracy and better meet the needs of the population. This was also consistent with our previous results between Ningxia, Shanxi, and Shenzhen. In our previous iodine balance studies, the 63.4 and 47.0 μg/d were the minimum iodine requirement of young males from Ningxia and Shenzhen [17, 18]. The results from iodine balance studies in Shanxi male adults also showed that when the iodine intake ranged from 16.3 to 134.4 μg/d, the estimated RNI of iodine for men (70.4 μg/d) was significantly lower than the current recommended intake. On the other hand, the iodine excretion was 48.6 μg/day at the first stage in this study. After 40 days of low iodine depletion, the 24-h UIC plateaued. In the first stage of the experiment, the increase of dietary iodine was 0, and the iodine excretion by urine and feces represented the necessary iodine excretion of people with normal thyroid function. The estimated EAR was 52.2 μg/d in this study and was higher than this iodine excretion, which indicated the recommended dose could meet people's basic iodine needs. Although the difference between classic iodine balance and 'iodine overflow theory' needs further study, the 'iodine overflow theory' provides a new idea for iodine balance research.

At present, there is no significant difference in the recommended intake of iodine between sexes [13]. But thyroid diseases are more likely to occur among females than males in adulthood [32–34]. Epidemiological data have revealed direct effects of estrogen on thyroid function, which can enhance the iodine accumulation ability of thyroid follicles, promote the proliferation of thyroid cells, and promote the synthesis of thyroid hormone, thus increasing the demand for iodine [35, 36]. The research results between male and female adults from Shanxi iodine balance studies showed that female adults needed more iodine intake than male adults in the same iodine intake range. The EAR and RNI were estimated at 47.8 μg/day (0.7 μg/d kg) and 70.4 μg/day (1.1 μg/d kg) in male adults from Shanxi iodine balance studies. There was little difference in the recommended dose per day (47.8 μg/day vs. 52.2 μg/d, 70.4 μg/day vs. 73.1 μg/d), but from the perspective of iodine requirement per kilogram of body weight, the requirement of female adults was more than 20% higher than that of male adults (0.7 μg/d kg vs. 1.0 μg/d kg, 1.1 μg/d kg vs. 1.4 μg/d kg). The results in Ningxia and Shenzhen also showed that when female adults reach iodine balance, the intake of iodine may be higher [17, 18]. In addition to the influence of hormones and menstrual cycle, it may be due to the different needs for iodine caused by the metabolic differences between different sexes, and the difference needs further study.

The present study has both strengths and weaknesses for deriving a daily iodine requirement based on balance studies. First, strict quality control was used to ensure the level of iodine intake and the integrity of sample collection. At the same time, accurate and precise analytic methods are key factors for iodine balance experiment. In our study, we used ICP-MS and external quality controls, and the results showed high precision and low CVs. Second, our sample contained six stages and its size was a relatively large sample size in relevant balance studies [10–12, 31, 37]. The experimental period was also longer than previous studies and we took into account the menstrual periods of women, and used consistent diet and urinary iodine monitoring to exclude the influence of menstrual periods. Third, we underestimated the iodine excretion in this study because iodine excretion from sweat, skin, and other source was not measured. More studies are needed to explore iodine requirement in Chinese female adults.

In conclusion, by excluding the influence of menstrual periods, we studied the iodine requirement of Chinese female adults based on ‘iodine overflow theory’. According to this theory, iodine intake of 52.2 μg/d (1.0 μg/d kg) may meet the minimum iodine requirement for Chinese female adults, and Chinese female adults may need more than 20% iodine intake than male adults. Our study provided a scientific evidence base for a reasonable reference intake of iodine in Chinese female adults. However, there was a great difference between the existing standard and our finding. Further studies are required to verify the results and the theory.

## Data Availability

The data of anonymized subjects are available for use in collaborative studies to researchers upon reasonable request to the corresponding author (xgyangcdc@163.com). The relevant data will be provided following the scientific review and formal approval of a research proposal (including a detailed experimental protocol and a statistical analysis plan) and completion of a data sharing agreement. Responses to the request for the raw data will be judged by a committee including YXG, LXL and GCZ.
